# Supplementary material for: A proteome-wide quantitative platform for nanoscale spatially resolved extraction of membrane proteins into native nanodiscs
Source: bioRxiv. 2024 Mar 12:2024.02.10.579775. Originally published 2024 Feb 12. Preprint. [Version 2] doi: 10.1101/2024.02.10.579775 (PMC10888908; doi:10.1101/2024.02.10.579775)
Supplement: Supplementary file 1 [file media-1.pdf]

Supplementary Information Table 1: Structures solved using membrane active polymers

Protein	Polymer	Year	Purified From	Resolution	Reference
ACRB	SMA 2:1	2018	E. coli	8.8 Å	[40]
ACIII	SMA 3:1	2018	F. johnsoniae	3.4 Å	[15]
ZipA:FtsZ	SMA 2:1	2019	E. coli	16 Å	[41]
KimA	SMA 2:1	2020	E. coli	3.7 Å	[42]
hTRPM4	AASTY-B	2020	HEK293	18 Å	[18]
BdSLAC1	SMA (not specified which type)	2021	S. pombe	2.97 Å	[43]
Cyt bo_3	SMA 3:1	2021	E. coli	2.55 Å	[44]
ELIC	SMA 3:1	2021	E. coli	2.5 Å	[45]
Yna1	SMA 2:1	2021	E. coli	2.4 Å	[46]
GlyR	SMA2:1	2021	Sf9	3.2 Å	[47]
Bam complex	SMA 2:1	2022	E. coli	3.6 Å	[48]
cASIC1	SMA 2:1	2020	HEK293S GnTI	2.8 Å	[49]
HIV-1 Env	SMA 2:1	2023	A549	4.1 Å	[50]
WbaP	SMA 2:1	2023	E. coli	Not specified	[51]
Cyt bc_1	SMA (not specified which type)	2023	R. sphaeroides	2.9 Å	[52]
GPIb-1X-V	SMA 3:1	2023	Expi293F	11 Å	[53]

Supplementary Information Table 2: Polymer description

Polymer Name	Description	Synthesis Methods	Origin
SMA140	Styrene, Maleic Acid ratio 1:1; MW 6.5 KDa	CSTR	Commercial
SMA200	Styrene, Maleic Acid ratio 2:1; MW 6.5 KDa	CSTR	Commercial
SMA300	Styrene, Maleic Acid ratio 3:1; MW 6.5 KDa	CSTR	Commercial
AASTY645	Styrene, Acrylic Acid ratio 55:45; MW 6 KDa	RAFT	Commercial
AASTY650	Styrene, Acrylic Acid ratio 50:50; MW 6 KDa	RAFT	Commercial
AASTY655	Styrene, Acrylic Acid ratio 45:55; MW 6 KDa	RAFT	Commercial
AASTY1145	Styrene, Acrylic Acid ratio 55:45; MW 11 KDa	RAFT	Commercial
AASTY1150	Styrene, Acrylic Acid ratio 50:50; MW 11 KDa	RAFT	Commercial
AASTY1155	Styrene, Acrylic Acid ratio 45:55; MW 11 KDa	RAFT	Commercial
CS20	Chloro Styrene, Maleic Acid ratio 1:1; MW 6.0 KDa	RAFT	Home made
CS40	Chloro Styrene, Maleic Acid ratio 1:1; MW 6.6 KDa	RAFT	Home made
CS60	Chloro Styrene, Maleic Acid ratio 1:1; MW 7.7 KDa	RAFT	Home made
CS80	Chloro Styrene, Maleic Acid ratio 1:1; MW 8.8 KDa	RAFT	Home made
AASTY80	Styrene, Acrylic Acid ratio 55:45; MW 5.1 KDa	RAFT	Home made

Supplementary Information Table 3: Extraction conditions

Extraction Condition	Buffer Condition	Salt Concentration	10% glycerol	% polymer
SMA140	20 mM HEPES pH 7.5	100mM NaCl	N	1%
SMA200	50 mM TrisHCl pH 8.1	300mM NaCl	Y	1%
SMA300	20 mM HEPES pH 7.5	100mM NaCl	N	1%
AASTY645	20 mM HEPES pH 7.5	100mM NaCl	N	1%
AASTY650	20 mM HEPES pH 7.5	100mM NaCl	N	1%
AASTY655	20 mM HEPES pH 7.5	100mM NaCl	N	1%
AASTY1145	20 mM HEPES pH 7.5	100mM NaCl	N	1%
AASTY1150	20 mM HEPES pH 7.5	100mM NaCl	N	1%
AASTY1155	20 mM HEPES pH 7.5	100mM NaCl	N	1%
CS20	50 mM TrisHCl pH 8.1	150 mM NaCl	Y	1.5%
CS40	50 mM TrisHCl pH 8.1	150 mM NaCl	Y	1.5%
CS60	50 mM TrisHCl pH 8.1	150 mM NaCl	Y	1.5%
CS80	50 mM TrisHCl pH 8.1	150 mM NaCl	Y	1.5%
AASTY80	50 mM TrisHCl pH 7.8	150 mM NaCl	Y	1.5%
